# Breastfeeding Experiences of Mothers With Visual Impairment: A Scoping Review

**DOI:** 10.1111/mcn.70061

**Published:** 2025-06-29

**Authors:** Emma‐Rose Biggar, Lisa McKenna, Lisa H. Amir

**Affiliations:** ^1^ School of Nursing and Midwifery La Trobe University Bundoora Victoria Australia; ^2^ Judith Lumley Centre, School of Nursing & Midwifery La Trobe University Bundoora Victoria Australia; ^3^ Breastfeeding service Royal Women's Hospital Parkville Victoria Australia

**Keywords:** blindness, breastfeeding, lactation, mother, scoping review, support, visual impairment

## Abstract

Breastfeeding rates are lower for women with disabilities than for women without disabilities, and women with visual impairment may be discouraged from breastfeeding by health professionals or their families. Little is known about how women with visual impairments learn to breastfeed and their breastfeeding experiences. The aim of this scoping review was to identify and map available evidence regarding the experiences and needs of mothers with visual impairment in initiating, establishing and maintaining lactation and breastfeeding, as well as to examine the extent of existing knowledge regarding supports and services currently available to meet those needs. We searched the following: MEDLINE, CINAHL, Embase, Scopus, the Cochrane Database and JBI Evidence Synthesis, Directory of Open Access Journals (DOAJ) and ProQuest Dissertations and Theses. Grey literature sources were searched via Google Scholar. Initial searches, after removal of duplicates, title and abstract screening, resulted in 39 articles for full text screening, of which, 17 met the inclusion criteria. Data were analysed using thematic synthesis. Studies came from seven countries and were mostly qualitative. Two analytical themes were generated: Visual impairment increases women's difficulty in learning to breastfeed, and Peer support is essential for visually impaired mothers, as HCPs provide poor support for breastfeeding. Visual impairment adds an extra layer of difficulty when women are learning to breastfeed. Barriers are created when systems and structures did not accommodate a woman's inability to see. Lack of access to information in nonvisual formats, limited transport to attend appointments and unsupportive attitudes from healthcare workers were common.

## Summary

- The needs of women with visual impairment in relation to breastfeeding have not been widely explored.
- Low vision and blindness make learning to breastfeed more challenging.
- Health professionals require specific education to care for women with visual impairment.
- Supports that accommodate specific needs of women with visual impairment need to be developed.

## Graphical abstract

This scoping review explored the breastfeeding experiences of mothers with visual impairments. We found that having a visual impairment can increase difficulties in learning to breastfeed due to barriers like lack of accessible information and unsupportive healthcare attitudes, with peer support identified as essential, as health professionals often provided inadequate assistance.

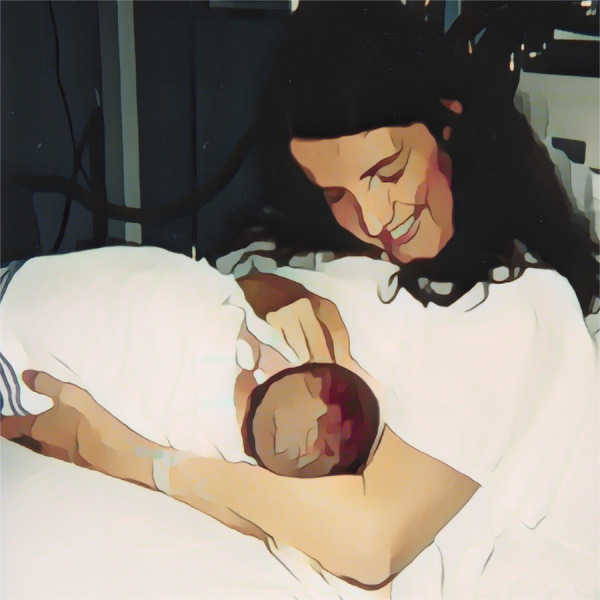

## 1 Introduction

Vision is the most dominant sense and forms an integral part in every facet and stage of one's life (World Health Organization 2019). Visual impairment is defined as a significant limitation of visual capacity and includes both those with low vision and/or blindness (Australian Institute of Health and Welfare 2019). Globally, moderate to severe vision impairment and blindness are estimated to affect around 4% of the population, many of whom are in their childbearing years (Bourne et al. 2021).

Breastfeeding rates for women with disabilities are lower than those of nondisabled peers (Malouf et al. 2017; Mitra et al. 2015; Morton et al. 2013; Ramer et al. 2024; Redshaw et al. 2013). Most women need practical help and support in learning how to breastfeed, regardless of whether they have a disability. Successfully establishing effective and comfortable breastfeeding can be heavily influenced by the support families receive. Duration and exclusivity have been reported to increase when women are offered breastfeeding support and counselling (Gavine et al. 2022). Without access to information and guidance, women are more likely to cease breastfeeding earlier than desired (Newby and Davies 2016).

Women with significant visual impairments experience a unique set of challenges navigating societies, systems and services built around the ability to see (World Health Organization 2019). While resources aimed to support families to establish breastfeeding and maintain lactation are available, it is unknown if women with visual impairment or blindness use these resources and how useful they find them. At present, little is known about how women with visual impairments learn the skill of breastfeeding and about their experiences as breastfeeding mothers. Hence, it is timely to explore the experiences and needs of this population to enable the identification and development of appropriate breastfeeding and lactation supports. This scoping review aimed to identify and map available evidence regarding the experiences and needs of mothers with visual impairment in initiating, establishing and maintaining lactation and breastfeeding, as well as to examine the extent of existing knowledge regarding supports and services currently available to meet those needs.

## 2 Methods

Scoping reviews map a broad range of literature with the aim to present an overview on a topic (Arksey and O'Malley 2005), so may contain a variety of resource types. This review was conducted in accordance with the Joanna Briggs Institute (JBI) guidelines methodology for scoping reviews (Peters et al. 2020). The methods used were guided by the Preferred Reporting Items for Systematic reviews and Meta‐Analysis extension for Scoping Reviews (PRISMA‐ScR) checklist (Tricco et al. 2018). The process of the review included stages of: identifying the research question, identifying relevant studies, selecting studies, extraction and charting the data (Arksey and O'Malley 2005).

### 2.1 Eligibility Criteria

Studies were evaluated for eligibility based on inclusion criteria structured according to population, concept, context (PCC) criteria (Peters et al. 2020). The *population* was women with visual impairment including complete or partial blindness, the *concept* of interest was experiences relating to breastfeeding and lactation and *context* was all countries and settings. No predefined criteria or definition of blindness was used; studies were included if the authors identified participants as having a visual impairment. Studies were included if they described women's experiences relating to breastfeeding, including breastmilk feeding and lactation. There were no date ranges or limits to geographical location applied. Only sources available in the English language were included in this review.

### 2.2 Search Strategy

An initial search of MEDLINE and Embase was undertaken to identify articles on the topic. Based on this, a full search strategy, including keywords and index terms, was developed in consultation with a university librarian and adapted for each database and information source as required (see Supporting information). These included MEDLINE, CINAHL, Embase, Scopus, Cochrane Database and JBI Evidence Synthesis, Directory of Open Access Journals and ProQuest Dissertations and Theses. Grey literature sources were searched via Google Scholar. In addition, tables of contents of journals specific to breastfeeding were hand searched (*Breastfeeding Medicine*, *Breastfeeding Review, International Breastfeeding Journal* and *Journal of Human Lactation*) and reference lists of identified studies were searched for any additional publications not previously identified. Searches were conducted in September 2022, with additional supplementary searches conducted in September 2023 and September 2024.

### 2.3 Source of Evidence Screening and Selection

**Figure 1:**
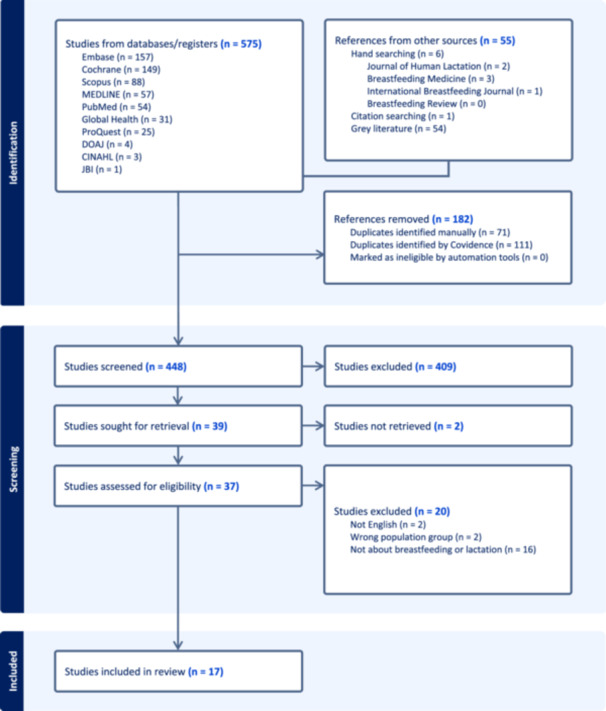
PRISMA‐ScR flowchart (Tricco et al. 2018).

Screening was conducted according to PRISMA‐ScR (Tricco et al. 2018) (Figure 1). From the initial search and additional supplementary searches, 630 records were identified (see Figure 1) which were uploaded into EndNote and imported to Covidence. Duplicates were removed (*n* = 182), leaving 448 records for title and abstract screening against the inclusion criteria. Reasons for exclusion were not recorded during the titles and abstracts screening stage, as Covidence does not collect reasons for exclusion at this stage. Of the 448 records screened, 39 proceeded to full text screening. All screenings were completed independently by two members of the research team. Where any conflict arose, these were resolved by a third researcher in collaboration with all team members. A total of 17 studies met the inclusion criteria and were included in the final review. Reasons for exclusion at full‐text screening were: did not include experiences related to breastfeeding or lactation (*n* = 16), reported experiences were not specific to participants with visual impairment (*n* = 2), articles not available in English (*n* = 2), full text not available (*n* = 2).

### 2.4 Data Extraction

A data extraction table was developed, items included first author; year of publication; country of study; aim; study design and data collection method; participants; results; study limitations; and quality assessment. Data were extracted by two authors and reviewed by a third.

Scoping reviews do not typically set out to assess the quality of included evidence (Tricco et al. 2018), however, critical appraisal was included during data extraction to assist in evaluating the quality of identified studies. This is intended to provide an overall assessment of the quality of available evidence and guide future research. Critical appraisal was not used for exclusion purposes at any stage of the review. Currently, there is no consistent approach recommended for conducting quality appraisals within scoping reviews. For the purpose of this review, the Critical Appraisal Skills Programme (CASP) tools were used as appropriate to the individual study design (Critical Appraisal Skills Programme 2018). The CASP Qualitative Studies Checklist (10 questions) was used to assess the included qualitative studies, and the CASP Checklist for Descriptive/Cross‐Sectional Studies (11 questions) was applied to the included studies with those designs. Each item was rated as ‘Yes’, ‘No’ or ‘Can't tell’, and a percentage score was calculated for each study based on the number of ‘Yes’ responses. A detailed summary of CASP scores for each study is provided in Supporting Information File S1.

### 2.5 Analysis and Presentation of Results

Selected sources of evidence were analysed using a thematic synthesis approach (Thomas and Harden 2008). This process involved three distinct stages: first, open coding ‘line‐by‐line’ through reading and rereading the included articles; second, development of descriptive themes from initial codes; and third, the generation of analytical themes that ‘go beyond’ the primary studies. In the final step, the descriptive themes were analysed in light of the aim of the review to generate new themes that account for patterns across the included studies. Codes and descriptive themes were discussed with all authors to provide diverse insights and interpretations of the data, facilitating the generation of analytical themes.

### 2.6 Ethics Statement

This scoping review synthesises existing literature and does not involve any direct interaction with human participants. Therefore, ethical approval was not sought, as the study relies solely on published data.

## 3 Results

### 3.1 Characteristics of Included Studies

Articles were published in Brazil (*n* = 3), Canada (*n* = 2), Ghana (*n* = 2), Greece (*n* = 1), Italy (*n* = 1), Malaysia (*n* = 1), Turkey (*n* = 2) and USA (*n* = 5) (see Table 1). Dates of publication ranged from 1996 to 2024, with most being published between 2020 and 2024 (*n* = 10). Most studies used qualitative designs (*n* = 15), with interviews being the primary source of data collection. Most (*n* = 14) focused specifically on women with visual impairment, others focused on disability as a broader topic and included women with a range of physical, cognitive and sensory disabilities. Details relating to onset of participants' visual impairments was discussed in ten of the studies, and description of level or type of impaired vision was provided in eight of the studies. Most participants in the studies were mothers (primiparas and multiparas), however some articles included pregnant women with visual impairments, health service workers, relatives of the woman with the disability and fathers with visual impairments. The number of participants in each included article ranged from 2 to 55 and reported age ranged from 18 to 72 years. Of the studies that provided details about children's ages, these ranged from several months of gestation to a 31‐year‐old (adult child).

**Table 1:** Characteristics of included publications.

Author, year of publication, country	Aim	Study design/data collection method	Participants, onset and level of vision	Results	Study limitations	Quality^a^
Acar et al. (2024), Turkey	To identify challenges faced by women with visual impairment during preconception, pregnancy, birth and the postpartum period	Qualitative Interviews	Total = 17 Onset – discussed, intext details do not match numbers presented in the table. In text – congenital (*n* = 15). In table – congenital (*n* = 2), ‘postnatal’ (*n* = 15) Level of vision – NA	− Concern reported regarding decision to have a child and social pressure on the decision to have a child reported − Inaccessible pregnancy tests, emotional reactions to pregnancy, dissatisfaction with healthcare providers, and unmet expectations − Mixed feelings about labour and birth, dissatisfaction with healthcare providers and unmet expectations − Postpartum period experiences of personal care, infant care, reaction of the social network, and unmet expectations described	Small sample size Participant selection, purposive sampling Self‐reported data Limited geographic diversity	100%
Acheampong et al. (2022), Ghana	To explore experiences of blind mothers as they navigated the process of motherhood	Qualitative Interviews	Total = 19 Onset – NA Level of vision – NA	− Difficulties experienced in areas of feeding, disciplining and protection. Challenges with poverty, discrimination, prejudices, psychological distresses such as depression also reported	Participant selection, purposive sampling Self‐reported data Small sample size Limited geographic diversity Not generalisable	90%
Andrews et al. (2021), USA	To explore lived experiences of disabled women related to breastfeeding	Qualitative Interviews	Total = 24 Physical (*n* = 10), sensory (*n* = 9), neurodevelopmental (*n* = 3), psychiatric or combination of these (*n* = 2) Onset – NA Level of vision – NA	− Communication difficulties with lactation consultants − Milk supply and latch problems − Intense pressure to breastfeed − Positive interactions with healthcare providers	Heterogeneity between and within cohorts Participant selection, convenience sampling Self‐reported data Small sample size of cohorts Not generalisable	100%
Bieber‐Schut (1993), Canada	To explore motherhood experiences for women with visual impairment	Qualitative Interviews (informal)	Total = 11^b^ Support group members (*n* = 9), with a father (*n* = 1) attending one of the sessions. The author includes her own experiences in the Discussion Onset – The women also experienced different eye conditions: juvenile macular degeneration, retinopathy of prematurity, retinitis pigmentosa, glaucoma, diabetic retinopathy, and detached retinas Level of vision – ‘visual loss for each woman was of varying degrees: no light perception, light perception only, legal blindness (an acuity of 20/200 or less in the best eye after all possible correction, or a visual field of less than 20 degree), or low vision (an acuity of greater than 20/200)’	− Prenatal classes were insufficient − Breastfeeding was a frustrating challenge made harder by visual impairment − Many ended up formula feeding due to lack of support and frustration − Mixed experiences of support from hospital staff both good and bad − Transport limitations impacted access to services	Poor methodology transparency No defined data analysis Researcher bias not addressed Unclear sample size Self‐reported data Small sample size Participant selection, purposive sampling Non‐validated tool for analysis used Not generalisable	40%
Buor et al. (2022), Ghana	To explore challenges facing women with disability in exclusively breastfeeding their infants	Qualitative Interviews; Focus group	Total = 55 Participants with disabilities (*n* = 45); physical (*n* = 20), deaf (*n* = 18), blind (*n* = 6), cerebral palsy (*n* = 1). Health service workers: midwives (*n* = 2), paediatrician (*n* = 1). Relatives of the women with a disability (*n* = 7); mothers (*n* = 4), husband (*n* = 2), cohabiter (*n* = 1) Onset – NA Level of vision – NA	− Health as a limiting factor which included inadequate nutrition, inadequate breastmilk, environmental‐induced conditions − Institutional constraints on disabled women included poor attitudes of health and communication by professionals − Physical conditions contributing to difficulty included caesarean section and incapacitation, not seeing mouths of infants to breastfeed − Family members can be discouraging of exclusive breastfeeding and husbands support may not be forthcoming. − Menial jobs of disabled women discussed in relation to financial constraints, inadequate feeding and production of milk	Heterogeneity between and within cohorts Participant selection, purposive sampling Not generalisable Self‐reported data Not generalisable	90%
Can and Mizrak Sahin (2023), Turkey	To examine breastfeeding experiences of mothers with visual impairment, as well as factors that facilitate and prevent these mothers from continuing to breastfeed	Qualitative Interviews	Total = 15 Onset – congenital (*n* = 11) acquired (*n* = 3), congenital right eye/left eye acquired (*n* = 1) Level of vision – 100% (*n* = 5), 95% (*n* = 2), 90% (*n* = 2), 85% (*n* = 1), 70% (*n* = 3), 50% (*n* = 1), 44% (*n* = 1)	− Negative emotions caused by breastfeeding − Barriers and facilitators to initiating and maintaining breastfeeding discussed	Self‐reported data Participants selection, snowball sampling recruitment Small sample size Limited geographic diversity Not generalisable	100%
Cezario et al. (2016), Brazil	To understand experiences of blind parents in care related to breastfeeding and complementary feeding of children	Qualitative Interviews	Total = 9 Men (*n* = 5) and women (*n* = 4) Onset – congenital (*n* = 5), acquired (*n* = 4) Level of vision – NA	− Breastfeeding and complementary feeding offered by blind mothers, insecurity in infant care and general challenges to establishing breastfeeding resulted in early cessation − Fathers can provide psychological support to the mother which helps facilitate the process of breastfeeding − Felt unprepared for childcare, felt excluded from social support networks − Coping mechanisms were developed to accommodate physical limitations	Participant selection, snowball sampling Small sample size Not generalisable Self‐reported data Limited geographic diversity	90%
Colaceci et al. (2023), Italy	To explore experiences and expectations of women with vision impairments regarding childbearing age, pregnancy, and motherhood	Qualitative Interviews	Total = 12 Onset – NA Level of vision – NA	− Stigma and communication gap experienced from healthcare professionals − Stigma surrounding sexuality − Emotions about motherhood include fears about pregnancy, delivery, and child health as well as resilience − Seeking information and decision‐making during pregnancy discussed − Support received during hospital stay, post‐discharge	Participant selection, purposive sampling Self‐reported data Inclusion criteria not defined Not generalisable Small sample size Meanings may be lost in translation (quotes translated from Italian)	90%
Conley‐Jung (1996), USA	To explore issues relevant to mothers with visual impairment; identify sources of concern; assess the mothers access to social support and resources during early parenthood	Qualitative Interviews Questionnaire	Total = 42 Onset – ‘majority diagnosed between birth and age two’ Level of vision – 20/600 or below (*n* = 18), 20/400‐20/599 (*n* = 12), 20/200‐20/399 (*n* = 10), 20/50‐20/199 (*n* = 2)	− Some mothers prepared in advance and others learnt through trial and error − Reactions to pregnancies and parenting were generally positive and stable over time − A significant correlation existed between couple satisfaction and partners' support − Parenting with visual impairment required extra time, effort, and expense − Mothers valued connections with other visually impaired parents, even briefly − Participants found creative solutions to parenting challenges, and visual impairment did not diminish their parenting quality	Participant selection, purposive sampling Not generalisable Limited geographic diversity Self‐reported data	80%
Dias et al. (2018), Brazil	To evaluate breastfeeding self‐efficacy among blind mothers	Quantitative Interviews; (semi structured interview to collect demographic info) questionnaire (The Breastfeeding Self‐Efficacy Scale‐Short Form)	Total = 10 Onset – congenital (*n* = 7), acquired (*n* = 3) Level of vision – NA	− Mothers in the study had both high and low breastfeeding self‐efficacy	Small sample size Participant selection, convenience sampling/snowball Not generalisable Limited geographic diversity Self‐reported data	88%
Frederick (2015), USA	To examine instances of discrimination that blind mothers experience with doctors, nurses, and social workers during hospital postnatal care	Qualitative Interviews; Focus groups	Total = 26^c^ Onset – blind since birth (*n* = 13), lost sight during childhood/adolescence (*n* = 3), progressively lost (*n* = 9), sudden loss (*n* = 1) Level of vision – ‘some usable vision’ (*n* = 9), totally blind or have ‘light only perception’ (*n* = 17)	− Participants identified networks of blind mothers as the best source of information on alternative techniques − Medical professionals rarely provided information about alternative baby care methods for blind parents − Some are discouraged from breastfeeding due to their blindness	Not generalisable Participant selection, purposive sampling Researcher bias not addressed Claims made not supported by evidence Poor methodology transparency Inconsistencies in presented data Limited geographic diversity Self‐reported data	30%
Jackson (2020), USA	To identify and describe the physical and attitudinal barriers experienced by blind women during the perinatal period	Qualitative Interviews; Questionnaire	Total = 7 Onset – NA Level of vision – All participants described themselves as blind (without usable vision)	− Information remain difficult to obtain for blind mothers − Challenges related to physical movement and accessibility − Fear of losing custody of their baby − Comradery among blind mothers − Stigma from healthcare providers assuming blind women cannot safely care for their infants, results in decreased help‐seeking behaviours	Participant selection, purposive sampling Small sample size Limited geographic diversity Not generalisable Self‐reported data	90%
Makeroufa and Diamanti (2024), USA	To highlight importance of developing guidelines that improve healthcare quality, proficiency of HCP, and the availability of adequate facilities, as derived from the antenatal population with any degree of visual impairment experiences during pregnancy, childbirth, and puerperium	Qualitative Questionnaire	Total = 22 Onset – congenital (*n* = 14), acquired (*n* = 8) Level of vision – totally blind (*n* = 14), partially blind (*n* = 8)	− Healthcare providers need training on the specific needs of visually impaired patients − Specialised resources for visually impaired women are needed, including individualised care plans − Policy interventions recommended for equitable healthcare access − Monitor mental health and provide appropriate referrals and support − Peer‐led support networks for visually impaired parents should be encouraged	Limited geographic diversity Small sample size Not generalisable Participant selection, purposive sampling Self‐reported data	80%
Maryam (2024), Malaysia	To provide an insight into the information needs of first‐time mothers with visual impairments as they embrace motherhood	Qualitative Interviews Audio diary	Total = 5 Onset – congenital (*n* = 3), acquired (*n* = 2) Level of vision – blindness in both eyes (*n* = 2), total blindness in right eye and slight vision in left (*n* = 1), total blindness in both eyes and slight vision in light (*n* = 1), total blindness in both eyes and slight vision in light and shape (*n* = 1)	− Information needs included baby care, mother care, family welfare, daily activities, and rights for people with disabilities	Small sample size Participant selection, snowball sampling Not generalisable Limited geographic diversity Self‐reported data	100%
Pagliuca et al. (2009), Brazil	To explore experiences of blind parents caring for their children	Qualitative Interviews	Total = 2 A mother (*n* = 1) and a father (*n* = 1) Onset – NA Level of vision – NA	− Parents with visual impairment face difficulties in taking care of their children − Parents with visual impairment develop adaptive strategies to take care of their children	Small sample size Not generalisable Participant selection, purposive sampling Self‐reported data Limited geographic diversity	70%
Shackelford (2004), USA	To explore blind mothers' perceptions of the impact of their blindness as it relates to interactions with their young child and with their parenting experiences	Qualitative Interviews	Total = 7 Onset – NA Level of vision – ‘Totally blind mothers or mothers with minimal light perception’	− Influences on parenting experiences can be internal and external − Support systems for blind mothers important − Modifications made between mother and child to facilitate communication and activities	Small sample size Not generalisable Participant selection, purposive sampling Self‐reported data Limited geographic diversity	100%
Tarasoff et al. (2023), Canada	To understand formal postpartum care experiences of birthing people with disabilities, including care for themselves and their newborns	Qualitative Interviews	Total = 31: physical (*n* = 14), sensory (*n* = 7), intellectual/developmental (*n* = 6), multiple disabilities (*n* = 4) Onset – NA Level of vision – NA	− Participants, experience of postpartum care was insufficient, HCPs lacked awareness of disability and accommodations − Fear of judgement, discrimination, and intrusive surveillance common experience	Unequal number of participants in each group Heterogeneity between and within cohorts Participant selection, purposive sampling Not generalisable Self‐reported data Limited geographic diversity	100%

Abbreviations: HCP = healthcare provider; NA = not available.

^a^ Value based on % of yes answers (see Supporting File S1: CASP quality assessment checklist).

^b^ It is not clear if the author counted herself as a group member.

^c^ Total number reported is contradictory to other data presented.

### 3.2 Review Findings

Ten descriptive themes were identified from the experiences reported in the included studies. From these, two analytical themes were generated: *visual impairment increases women's difficulty in learning to breastfeed;* and *peer support is essential for mothers with visual impairment as health care professionals provide poor support for breastfeeding*. An overview of the analytic themes and descriptive themes is provided in Table 2 and a more detailed description is presented in the next section.

**Table 2:** Charting the data – Thematic synthesis.

Analytic themes	Descriptive themes	Codes
Visual impairment increases women's difficulty in learning to breastfeed	*General breastfeeding challenges* ‘Breastfeeding, in particular, seemed to be one of the most frustrating and hopeless obstacles’	Learning to breastfeed was frustrating and stressful
		Breast and nipple pain
		Latching difficulties
		Milk supply concerns
		Using a formula to alleviate anxiety
		Low breastfeeding self‐efficacy
		Frustration and shame from inability to breastfeed
	*Breastfeeding success* ‘My breastfeeding role as a mother is one of the most joyous experiences’	Successful breastfeeding established
		Breastfeeding as an opportunity to bond
		Relying on other senses
		Pride in resourcefulness/ability to access information independently
	*Visual component to learning how to breastfeed* ‘To make matters worse, there seems to be a major visual component in the breastfeeding process’	Visual impairment added extra barrier to learning to breastfeed
		Difficulty using lactation aids
	*Lack of appropriate resources* ‘There were also no resources specific to the needs of blind mothers’	Resources not specific to needs of mothers with visual impairment
		Breastfeeding information provided in inaccessible visual formats
	*Transport limitations* ‘Mobility has always been difficult, and with a young baby, it is just that much harder’	Transport restrictions limited access to support services
		Mother‐baby separation
Peer support essential for visually impaired mothers as HCPs provide poor support for breastfeeding	*Inadequate training of HCP* ‘Sometimes the way people think they're helping is not the way that we really need that help’	Negative experience with HCP
		HCP inadequately trained
		Support offered was insufficient
		Support did not accommodate visual impairment
		Feeling excluded during consultations
	*Judgment* ‘Well, you might just really want to consider bottle feeding because … I just don't know how you're going to latch him on ever’	Felt judged and assumed incompetent because of disability
		Discouraged from breastfeeding
	*Effective breastfeeding support* ‘Only sheer determination and the supportive knowledge of a breastfeeding consultant … led me to success’	Breastfeeding support from HCP
	*Friends and family* ‘Thankfully, my neighbor became our eye’	Friends and family provided practical assistance
		Mother‐to‐mother support groups felt inclusive
	*Peers* ‘Best advice was from those other moms that have walked through that territory’	Shared experience of living with visual impairment
		Other parents with visual impairment shared own adaptations

Abbreviation: HCP = healthcare provider.

#### 3.2.1 Visual Impairment Increases Women's Difficulty in Learning to Breastfeed

The first analytical theme *visual impairment increases women's difficulty in learning to breastfeed* combines five descriptive themes: *general breastfeeding challenges, breastfeeding success, visual component to breastfeeding, lack of appropriate resources* and *transport limitations*. These descriptive themes highlight the complexities that mothers with visual impairment faced when learning to breastfeed. Many of the common challenges such as latching issues, nipple pain and concerns regarding milk production were reported. However, for these mothers having a vision impairment added an additional layer of complexity in learning to breastfeed and accessing supports and resources.

##### 3.2.1.1 General Breastfeeding Challenges

In included studies, women with visual impairment described learning to breastfeed as frustrating and stressful (Andrews et al. 2021; Bieber‐Schut 1993; Shackelford 2004). Women spoke of surprise in learning that knowing how to breastfeed was not automatic, which was unexpected (Bieber‐Schut 1993; Shackelford 2004). ‘*Breastfeeding, in particular, seemed to be one of the most frustrating and hopeless obstacles’* (Bieber‐Schut 1993, 3).

Reported challenges included breast and nipple pain (Acheampong et al. 2022; Can and Mizrak Sahin 2023; Cezario et al. 2016; Nurul and Corresponding Author 2024), latching difficulties (Acar et al. 2024; Acheampong et al. 2022; Bieber‐Schut 1993; Can and Mizrak Sahin 2023; Frederick 2015; Jackson 2020; Nurul and Corresponding Author 2024; Pagliuca et al. 2009), worry about milk production or supply concerns (Can and Mizrak Sahin 2023; Cezario et al. 2016; Shackelford 2004) as well as difficulty in finding a comfortable position and hold (Acar et al. 2024; Can and Mizrak Sahin 2023). Some women reported using infant formula as a method to measure their babies' intakes, which eased anxiety about low supply (Can and Mizrak Sahin 2023). ‘*After I had her drink her milk with a bottle, I felt comfortable saying ‘I fulfilled my responsibility, her stomach is full now’ and I was confident*’ (Can and Mizrak Sahin 2023, 546).

Low self‐efficacy and feelings of insecurity or lacking self‐confidence in ability to breastfeed was identified in six studies (Can and Mizrak Sahin 2023; Cezario et al. 2016; Dias et al. 2018; Nurul and Corresponding Author 2024; Pagliuca et al. 2009; Shackelford 2004). Women spoke of concerns about not only their ability to breastfeed successfully, but also whether they would be able to learn the skill of breastfeeding or recognise their babies' hunger cues. Additionally, women spoke about discomfort and insecurity about breastfeeding in public for fear of being stared at (Shackelford 2004). Interruption to breastfeeding and early cessation was discussed in four of the studies (Andrews et al. 2021; Bieber‐Schut 1993; Cezario et al. 2016; Dias et al. 2018). When breastfeeding either did not work out or meet women's expectations, some reported feelings of frustration, anger and shame (Andrews et al. 2021; Bieber‐Schut 1993; Can and Mizrak Sahin 2023).

I had a meltdown. I felt that somehow breastfeeding should've been the one thing I should've been able to do easily. And at the prospect of not breastfeeding him, I felt like the one thing I knew I could do with no problem as a blind mother suddenly became a problem.

(Andrews et al. 2021, 96)

I just couldn't kill the feeling of incompetence. I'll just hold the breast and breastfeed. But I couldn't do it, I failed, there was something about me. And I was feeling inadequate.

(Can and Mizrak Sahin 2023, 544)

##### 3.2.1.2 Breastfeeding Success

Despite initial challenges, some women reported success in learning how to breastfeed: ‘*… I learned to breastfeed over time… then I started to give other things, fruits, mash, I made it myself…’* (Pagliuca et al. 2009). Further, women spoke of strategies to know when to feed their babies, relying on their other senses and learning to breastfeed tacitly (Colaceci et al. 2023; Conley‐Jung 1996; Pagliuca et al. 2009; Shackelford 2004).

At first, I also struggled with touch to identify the position of the baby's lips when latching on, but I was working hard because I wanted to breastfeed. I pumped the milk manually, and the midwives explained how to do it and when to use the breast pump.

(Colaceci et al. 2023, 5)

High breastfeeding self‐efficacy was mentioned in one study (Dias et al. 2018). In two studies, women expressed joy and pride in being able to breastfeed and found breastfeeding to be an opportunity to bond with their babies (Acheampong et al. 2022; Can and Mizrak Sahin 2023).

My breastfeeding role as a mother is one of the most joyous experiences. I am able to locate the mouth of my child without any help and my ability to perform this role is what gives me a sense of fulfilment and affirmation as a mother.

(Acheampong et al. 2022, 5)

##### 3.2.1.3 Visual Component to Learning How to Breastfeed

Reported experiences highlighted challenges specific to mothers with visual impairment. Participants shared examples of how their inability to rely on sight impacted their abilities to breastfeed, noting there was a significant visual component involved in learning how to breastfeed. This added an additional barrier to establishing effective breastfeeding.

To make matters worse, there seems to be a major visual component in the breast‐feeding process. It was one thing to not know what to do, it was another to not be able to see and not know what to do. Heaven help you if you had an infant who refused to latch on. The stress could be enormous.

(Bieber‐Schut 1993, 3)

Some mothers reported challenges in finding comfortable positions, which was compounded by their inability to see: ‘*I had a lot of pain because I was trying to see*’ (Can and Mizrak Sahin 2023, 545). Some felt their visual impairment made it difficult to ensure proper alignment, leading to frustration and anxiety (Acar et al. 2024; Can and Mizrak Sahin 2023). Others discussed struggles in finding their babies' mouths and being able to direct their infants to the nipple. Additionally, some described worry that misalignment may lead to suffocation if their breast obstructed the infant's nose (Acar et al. 2024; Acheampong et al. 2022; Can and Mizrak Sahin 2023).

I'm not good with breastfeeding, don't have the skills to do it. I struggled a lot! I have experienced that when my baby cries for milk, I need to find her mouth with one hand, while the other hand is used to support her body. It gets me confused again, it's pressure.

(Nurul and Corresponding Author 2024, 11)

Some mothers mentioned difficulty in using lactation aids such as nipple shields and breast pumps: ‘*We bought a pump, I think it was to bring the nipple out. I tried to use it for milking. I cracked my nipple really bad because I didn't see what was going on’* (Can and Mizrak Sahin 2023, 545).

##### 3.2.1.4 Lack of Appropriate Resources

Information communicated to women across the perinatal period from antenatal classes to hospital admissions and following discharge was predominantly in visual formats, such as print. This created a barrier as women were not able to access the information due to their inability to see and read the printed content (Can and Mizrak Sahin 2023; Jackson 2020; Shackelford 2004). This was especially relevant for women needing breastfeeding information: ‘*… We came home with a big pocket, a big folder of printed material that we couldn't read’* (Jackson 2020, 77). Although some women discussed instances of being provided with resources in braille, this was not a common practice (Jackson 2020). Even when a mother was able to source assistance of a sighted friend or relative to read the information, the resources were identified as not specific to the needs of women with visual impairments (Acar et al. 2024; Frederick 2015; Jackson 2020; Shackelford 2004; Tarasoff et al. 2023).

There were also no resources specific to the needs of blind mothers. All of the resources and classes were geared toward sighted mothers.

(Shackelford 2004, 120)

##### 3.2.1.5 Transport Limitations

Transport restriction was discussed in three studies (Bieber‐Schut 1993; Jackson 2020; Shackelford 2004), reduced access to transport and restricted mobility impacting mothers' abilities to access support services (Bieber‐Schut 1993; Jackson 2020; Shackelford 2004), as well as contributing to the separation of mother and baby (Jackson 2020).

I said, I am blind, I cannot come every day and go back and come back. It's difficult for me. Plus, it's my only baby; I don't want to be separated from him. I want to be around my baby…

(Jackson 2020, 86)

#### 3.2.2 Peer Support Is Essential for Mothers With Visual Impairment as Healthcare Professionals Provide Poor Support for Breastfeeding

The second analytical theme, *peer support is essential for mothers with visual impairment as healthcare professionals provide poor support*, brings together five descriptive themes: *inadequate training of health care professionals, judgement, effective breastfeeding support, friends and family*, and *peers*. This theme illustrates a broader pattern: that mothers with visual impairment often faced inadequate, judgemental or ineffective support from healthcare professionals regarding breastfeeding. While some received helpful support from lactation consultants and midwives, practical assistance was more commonly found through friends, family and community groups. However, the most valuable guidance came from other parents with visual impairments, who shared lived experiences and could offer tailored strategies to navigate breastfeeding challenges.

##### 3.2.2.1 Inadequate Training of Healthcare Professionals

Both positive and negative interactions with healthcare professionals were identified throughout the included studies; however, the majority of reported experiences tended to be negative (Andrews et al. 2021; Bieber‐Schut 1993; Frederick 2015; Jackson 2020). In seven studies, women reported feeling that hospital and medical staff were inadequately trained or experienced in working with families with impaired vision (Andrews et al. 2021; Bieber‐Schut 1993; Can and Mizrak Sahin 2023; Colaceci et al. 2023; Frederick 2015; Jackson 2020; Tarasoff et al. 2023). Some women felt that health professionals' lack of knowledge or understanding resulted in missed opportunities for appropriate support.

I had a lot of difficulties with getting them to understand when they were explaining stuff. They were telling me like right here, over there. I had to keep reiterating, “I'm visually impaired. So by saying right here or right there, that is not going to help me. Can you please say go to your left, go to you right, move at 6 o'clock, move at 12 o'clock?” I felt that they were not trained on specifically how to interact with a blind mom.

(Andrews et al. 2021, 85)

In many studies, women discussed experiences whereby help offered was inadequate (Acar et al. 2024; Bieber‐Schut 1993; Can and Mizrak Sahin 2023; Colaceci et al. 2023; Frederick 2015; Makeroufa and Diamanti 2024; Nurul and Corresponding Author 2024; Tarasoff et al. 2023):

When you're in the hospital and they're teaching you how to breastfeed, they do this thing where they hold the baby, they wait until the baby's mouth is open, and then they fly it on when they think it's the right moment. And that doesn't teach the blind person how to breastfeed at all. That makes the blind person totally dependent on having the nurse there to watch when the baby's mouth is open.

(Tarasoff et al. 2023, 3330)

Women felt there were assumptions that they would figure out how to access information provided in print and other visual formats without further consideration to whether this was practical or appropriate, as described in one study: ‘*Overall, the participants seemed to believe that the burden of accessing the information fell on them instead of on the provider’* (Jackson 2020, 80).

##### 3.2.2.2 Judgement

More significantly, many reported incidents of feeling judged and stereotyped with presumption of incompetence because of their disability (Bieber‐Schut 1993; Cezario et al. 2016; Frederick 2015; Jackson 2020). Ordinary problems were described as incorrectly attributed to their vision impairment; however, later identified as not blindness related at all (Jackson 2020, 113–114). Some reported feeling they were not given equal standards of care because of assumptions or being excluded during consultations.

I'm not saying this to disrespect anyone, [but] they treat us like we're mentally handicapped. For example, let's say I'm going to breastfeed my baby and there is someone else with me. They explain the breastfeeding education to the person next to me.

(Can and Mizrak Sahin 2023, 545)

Furthermore, women reported being discouraged from breastfeeding by health professionals on the basis of their blindness, being advised against initiating breastfeeding and suggested to bottle (formula) feed instead (Frederick 2015).

We were down on the postpartum floor and the nurse helped me get him latched on but then she said, "Well, you might just really want to consider bottle feeding because… I just don't know how you're going to latch him on ever. Because I just saw that his mouth was open, and I just put the nipple in there for him, and I just don't know how you're going to do that.

(Frederick 2015, 1137–1138)

Across many studies, women reported feeling they needed to work hard to battle presumed incompetence and prove themselves as having equal ability to care for their children (Bieber‐Schut 1993; Cezario et al. 2016; Jackson 2020; Shackelford 2004). Some reported having social worker and child protection service involvement, which they believed was due to assumptions that their visual impairment meant they would not be able to care for their babies (Frederick 2015; Jackson 2020).

Six participants (Amy P1, Faith P2, Ingrid P3, Isabelle P4, Kelly P5, Lily P6) expressed feeling fearful of social services and one participant (Amy P1) reported that the hospital social worker took steps to remove her baby. All but one participant received a social worker visit during their hospitalization…. Distrust of social services was a common sentiment among the study participants.

(Jackson 2020, 88)

This distrust resulted in reduced help‐seeking for fear of negative consequences or outcomes despite acknowledging that help would be beneficial and was wanted.

I don't want other people making assumptions that I'm either not understanding how to feed my baby, or not feeding my baby, or not wanting to feed my baby. You know, are we going to have a failure to thrive thing? And then, am I going to have a lot of people intruding on my house, making some judgments that I don't feel comfortable with? It just it adds a layer of stress that (pause). Hey, it's not that we don't want to get help. It's not that we don't see a problem. We want to find out what would really help the situation, but sometimes the way people think they're helping is not the way that we really need that help, and the assumptions people make is just (pause). You just don't know where that's going to go.

(Jackson 2020, 93)

Many studies provided examples from women about feeling pressured to breastfeed perfectly. This was discussed in relation to several aspects of parenting in addition to breastfeeding.

Just the apprehension of, you know, being a blind mom and giving birth to your first kid and expecting to be visited by a social worker and having them take your kid away… You know, and it's just terrifying. And even like with the breastfeeding, I just felt like, ‘Oh God, I gotta do this perfect the first time or they're gonna take my kid away.

(Frederick 2015)

##### 3.2.2.3 Effective Breastfeeding Support

Examples were provided of times when mothers received support and help from healthcare professionals (Bieber‐Schut 1993; Can and Mizrak Sahin 2023; Colaceci et al. 2023; Jackson 2020; Tarasoff et al. 2023). ‘*Only sheer determination and the supportive knowledge of a breastfeeding consultant … led me to success*’ (Bieber‐Schut 1993, 3). Women spoke of practical support such as having their babies placed on them or being directed with verbal descriptions of adjustments to positioning and hand placement as well as being provided information/resources in braille or audio (Can and Mizrak Sahin 2023).

##### 3.2.2.4 Friends and Family

In five studies, women discussed friends and family members providing practical support, which was gratefully received (Bieber‐Schut 1993; Can and Mizrak Sahin 2023; Jackson 2020; Nurul and Corresponding Author 2024; Pagliuca et al. 2009). Community breastfeeding support services provided feelings of acceptance to counter stigma (Shackelford 2004).

Amy again mentioned the need for acceptance from her La Leche support group. She described this organisation with its mothers as a “safe place” in which she felt accepted as a mother first and a blind woman second. She got much of her parenting information from them.

(Shackelford 2004, 128)

##### 3.2.2.5 Peers

The greatest source of information and support reportedly came from other visually impaired parents who were able to provide information and adaptations specific to their needs to accommodate limitations of having a visual impairment (Colaceci et al. 2023; Frederick 2015; Jackson 2020; Shackelford 2004). This included information regarding not only breastfeeding but also the use of lactation aids such as breast pumps (Jackson 2020). Their lived experiences allowed shared understandings (Can and Mizrak Sahin 2023; Jackson 2020, 95). ‘*I would say that a lot of the best advice I got was from those other moms that have walked through that territory’* (Can and Mizrak Sahin 2023; Jackson 2020, 95).

## 4 Discussion

This scoping review explored available evidence regarding experiences and needs of mothers with visual impairment in initiating, establishing and maintaining lactation and breastfeeding, and the extent of existing knowledge regarding supports and services currently available to meet those needs. The review highlighted that even when women are highly motivated to breastfeed, many still encounter challenges. Breastfeeding challenges discussed in the reviewed studies align with findings from larger systematic reviews exploring breastfeeding generally, these included breast and nipple pain, concerns regarding insufficient supply, and poor latching (Marshall et al. 2021), but were compounded by visual impairment.

Despite initial challenges, some women reported learning to breastfeed over time and expressed joy and pride in the bonding experiences. Access to early, skilled and sensitive support can help resolve breastfeeding challenges for most women (Gavine et al. 2022); however, women with visual impairment experienced extra layers of difficulty in accessing help. In the included studies, many women spoke of barriers that were not based on their capabilities, but rather on limitations imposed by the systems and structures around them. The social model of disability provides a lens through which these experiences can be understood, suggesting that disability arises not from an individual's impairment, but rather from societal barriers that restrict access or participation (Oliver 1990). In this context, barriers were created, not only when systems and structures failed to accommodate these mothers' inabilities to see, but also through judgemental attitudes and discriminatory practices. Lack of access to information in nonvisual formats, limited transport to attend appointments and unsupportive attitudes from healthcare workers were common barriers to access. Additionally, fear of judgement, discrimination and intrusive surveillance by care providers were consistent findings for women with disabilities (Tarasoff et al. 2023). These findings underscore the need for specifically tailored information resources, as well as a need for tailored health professional preparation to support women with visual impairments to achieve successful breastfeeding outcomes.

This review yielded a very small number of studies from a small number of countries, indicating that globally, there has been little focus on this group of women to date. Furthermore, most included studies were small qualitative studies, often poorly reported. The lack of quantitative research means the scale of issues remains unclear. While some studies have explored breastfeeding for women with disabilities (Brown et al. 2023a; Brown et al. 2023b; Powell et al. 2019), these have not focused on specific needs of those with visual impairment. This suggests a need for larger and more representative studies to evaluate the overall scale of breastfeeding challenges faced by women with visual impairments and their needs to enable the development of appropriate supports. Furthermore, it illustrates the larger, systematic situation whereby women with disabilities are poorly identified and managed within maternity services (Benzie et al. 2023a, 2023b).

It is commonplace for women with disabilities to be treated as a homogenous group. Even when categories such as physical, intellectual or mental health disabilities are considered, visual impairment will frequently be grouped with deafness under the label ‘sensory disability’, assuming homogeneity within this category (Brown et al. 2023b; Malouf et al. 2017). Three studies in this review included participants with visual impairment alongside various other disabilities. The study by Buor et al. (2022) met the inclusion criteria presenting breastfeeding experiences of women with sensory disability, including visual impairment, but did not provide examples or statements specific to participants with visual impairment, despite being part of this group. The lack of specific examples from participants with visual impairment made it unclear if reported findings were reflective of the entire ‘sensory disability’ group. The lack of detailed representation by grouping visual impairment with deafness diminishes the ability to capture unique challenges and experiences of this particular subgroup and underscores the need for more targeted research.

Moreover, oversimplification fails to acknowledge the diverse challenges faced by women across different types of disabilities. For instance, while Warkentin et al. (2021) explored breastfeeding positions and techniques for women with physical disabilities, and Powell et al. (2019) described adaptive parenting strategies, there remains a gap in understanding how women with visual impairments navigate similar challenges. A recent study by Douglass et al. (2023) highlighted the specific healthcare needs of women with intellectual disabilities, reinforcing the importance of tailored support and accessible resources across all disability groups. By recognising the diversity within populations, research and resources can ultimately enhance care for all mothers.

Our review identifies the need to enhance healthcare providers' skills and knowledge to deliver patient‐centred care for women with visual impairments. Research is urgently needed to understand the factors influencing women with visual impairment's intention to breastfeed to codesign effective support and educational resources.

### 4.1 Limitations

The study has acknowledged limitations. First, the geographical scope was not widespread and did not include research from regions such as Oceania, the Middle East, Eastern Europe and other parts of Sub‐Saharan Africa and Latin America, which may limit the understanding of the experiences of mothers with visual impairments across diverse cultural and healthcare contexts. Additionally, there is limited research in areas with the highest incidence of blindness, which, according to Bourne et al. (2021), is reported to be currently in South Asia, East Asia, and Southeast Asia. As a result, the study's findings may not fully capture the varied experiences of individuals with visual impairments worldwide. This lack of coverage also means there is insufficient information on how health systems, lactation consultant availability, and cultural norms affect these individuals' experiences. The studies examined were conducted between 1996 and 2024, meaning that older studies may not accurately reflect current healthcare settings and practices. Furthermore, the review was limited to studies published in English, which may have excluded valuable insights from studies available in other languages.

## 5 Conclusion

This scoping review sought to explore what is known about breastfeeding for women with visual impairment. It identified a small number of studies, highlighting that many of the breastfeeding challenges experienced by these women are largely similar to those experienced by the general population. Women with visual impairment have additional unique challenges, much of which has not been explored in great depth. Based on the experiences presented, there is a need for further research to improve the delivery of information and breastfeeding support within the community and healthcare settings. In addition, further education for health professionals is needed, as well as support that accommodate and address specific breastfeeding needs that come with visual impairment.

## Author Contributions

Emma‐Rose Biggar drafted the protocol. Lisa H. Amir and Lisa McKenna revised the protocol. Emma‐Rose Biggar and Lisa McKenna screened titles, abstracts and full texts against inclusion criteria. Lisa H. Amir resolved any disagreements. Emma‐Rose Biggar and Lisa H. Amir extracted data. Emma‐Rose Biggar drafted the manuscript. Lisa H. Amir and Lisa McKenna revised the manuscript. All authors read and approved the final manuscript.

## Conflicts of Interest

The authors declare no conflicts of interest.

## Supporting information

Supplementary file 1 CASP quality assessment checklist.

Supplementary file 2 Medline search strategy.

Supplementary file 3 Embase search strategy.

## Data Availability

The data that supports the findings of this study are available in the supporting material of this article.
