# Supplementary material for: Breastfeeding Experiences of Mothers With Visual Impairment: A Scoping Review
Source: Matern Child Nutr. 2025 Jun 29;21(4):e70061. doi: 10.1111/mcn.70061 (PMC12454189; doi:10.1111/mcn.70061)
Supplement: Supplementary file 1 — CASP quality assessment checklist. [file MCN-21-e70061-s002.docx]

Critical Appraisal Skills Programme (CASP) quality appraisal

| Study number | First author, year of publication | Title |
| --- | --- | --- |
| 1 | Acar 2024 | Challenges faced by mothers with visual impairment from the preconception period through the postpartum period |
| 2 | Acheampong 2022 | Experiencing motherhood as a blind mother in the Greater Accra Region of Ghana; a qualitative study |
| 3 | Andrews 2021 | Experiences of Breastfeeding among Disabled Women |
| 4 | Bieber-Schut 1993 | Visually impaired women as mothers |
| 5 | Buor 2022 | Exploring the Challenges Facing Women with Disabilities in Exclusive Breastfeeding in a Metropolitan Area in Ghana: A Qualitative Study |
| 6 | Can 2023 | Experiences of Breastfeeding Mothers With Visual Disabilities |
| 7 | Cezario 2017 | Blind parents and nutrition of children: experiences and care |
| 8 | Colaceci 2023 | “They put the baby on me; the life I felt growing inside me was finally taking shape. Lived experiences of women with vision impairments during pregnancy and motherhood: A phenomenological qualitative study |
| 9 | Conley-Jung 1996 | The early parenting experiences of mothers with visual impairments and blindness |
| 10 | Dias 2018 | Breastfeeding self-efficacy among blind mothers |
| 11 | Frederick 2015 | Between stigma and mothers’ blame: Blind mothers' experiences in USA hospital postnatal care |
| 12 | Jackson 2020 | The Perinatal Experiences of Blind Women |
| 13 | Makeroufa 2024 | Exploring perinatal care and birth experiences in women with visual impairment: A retrospective study |
| 14 | Maryam 2024 | Information needs of a vulnerable community: The case of visually impaired first-time mothers in Malaysia |
| 15 | Pagliuca 2009 | Blind parents: their experience in care for their children |
| 16 | Shackelford 2004 | Blind mothers' perceptions of their interactions and parenting experiences with their sighted infants and toddlers |
| 17 | Tarasoff 2023 | Unmet needs, limited access: A qualitative study of postpartum health care experiences of people with disabilities |

*Note: # denotes corresponding study in qualitative appraisal tables*

CASP Checklist: For Qualitative Research

| **Check list questions** | **Study reference** | | | | | | | | | | | | | | |
| --- | --- | --- | --- | --- | --- | --- | --- | --- | --- | --- | --- | --- | --- | --- | --- |
|  | **1** | **2** | **3** | **4** | **5** | **6** | **7** | **8** | **11** | **12** | **13** | **14** | **15** | **16** | **17** |
| 1. Was there a clear statement of the aims of the research or publication? | **Y** | **Y** | **Y** | C | **Y** | **Y** | **Y** | **Y** | **Y** | **Y** | **Y** | **Y** | **Y** | **Y** | **Y** |
| 2. Is a qualitative methodology appropriate? | **Y** | **Y** | **Y** | **Y** | **Y** | **Y** | **Y** | **Y** | C | **Y** | **Y** | **Y** | **Y** | **Y** | **Y** |
| 3. Was the research design appropriate to address the aims of the research? | **Y** | **Y** | **Y** | **Y** | **Y** | **Y** | **Y** | **Y** | C | **Y** | **Y** | **Y** | **Y** | **Y** | **Y** |
| 4. Was the recruitment strategy appropriate to the aims of the research? | **Y** | **Y** | **Y** | **Y** | **Y** | **Y** | **Y** | **Y** | **Y** | **Y** | **Y** | **Y** | **Y** | **Y** | **Y** |
| 5. Was the data collected in a way that addressed the research issue? | **Y** | **Y** | **Y** | N | **Y** | **Y** | **Y** | **Y** | **Y** | **Y** | **Y** | **Y** | **Y** | **Y** | **Y** |
| 6. Has the relationship between researcher and participants been adequately considered? | **Y** | N | **Y** | C | C | **Y** | C | **Y** | N | C | C | **Y** | C | **Y** | **Y** |
| 7. Have ethical issues been taken into consideration? | **Y** | **Y** | **Y** | N | **Y** | **Y** | **Y** | **Y** | N | **Y** | **Y** | **Y** | **Y** | **Y** | **Y** |
| 8. Was the data analysis sufficiently rigorous? | **Y** | **Y** | **Y** | N | **Y** | **Y** | **Y** | **Y** | C | **Y** | **Y** | **Y** | C | **Y** | **Y** |
| 9. Is there a clear statement of findings? | **Y** | **Y** | **Y** | N | **Y** | **Y** | **Y** | **Y** | N | **Y** | **Y** | **Y** | **Y** | **Y** | **Y** |
| 10. How valuable is the research? Will the results help locally? | **Y** | **Y** | **Y** | C | **Y** | **Y** | **Y** | **Y** | C | **Y** | **Y** | **Y** | C | **Y** | **Y** |
| **Total # of yes answers** | **10** | **9** | **10** | **3** | **9** | **10** | **9** | **10** | **3** | **9** | **9** | **10** | **7** | **10** | **10** |

Y=yes, N=no, C=Can’t tell

CASP Checklist: For Descriptive/Cross-Sectional Studies

| **Check list questions** | **Study ref** | |
| --- | --- | --- |
|  | 9 | 10 |
| 1. Did the study address a clearly focused issue | **Y** | **Y** |
| 2. Did the authors use an appropriate method to answer their question? | C | **Y** |
| 3. Were the subjects recruited in an acceptable way? | **Y** | **Y** |
| 4. Were the measures accurately measured to reduce bias? | **Y** | **Y** |
| 5. Were the data collected in a way that addressed the research issue? | C | **Y** |
| 6. Did the study have enough participants to minimise the play of chance? | N | C |
| 7. How are the results presented and what is the main result? | **Y** | **Y** |
| 8. Was the data analysis sufficiently rigorous? | **Y** | **Y** |
| 9. Is there a clear statement of findings? | **Y** | **Y** |
| 10. Can the results be applied to the local population? | C | C |
| 11. How valuable is the research? | C | C |
| **Total # of yes answers** | **6** | **8** |

Y=yes, N=no, C=Can’t tell

Critical Appraisal Skills Programme. (2024a). *CASP Checklist: For Descriptive/Cross-Sectional Studies*. <https://casp-uk.net/casp-tools-checklists/cross-sectional-studies-checklist/>

Critical Appraisal Skills Programme. (2024b). *CASP Checklist: For Qualitative Research* <https://casp-uk.net/casp-tools-checklists/qualitative-studies-checklist/>
