# Supplementary material for: Breastfeeding Experiences of Mothers With Visual Impairment: A Scoping Review
Source: Matern Child Nutr. 2025 Jun 29;21(4):e70061. doi: 10.1111/mcn.70061 (PMC12454189; doi:10.1111/mcn.70061)
Supplement: Supplementary file 2 — Medline search strategy. [file MCN-21-e70061-s001.docx]

| **Medline** | |
| --- | --- |
| **#** | **Query** |
| 1 | (breastfeeding adj5 skill*).mp. [mp=title, book title, abstract, original title, name of substance word, subject heading word, floating sub-heading word, keyword heading word, organism supplementary concept word, protocol supplementary concept word, rare disease supplementary concept word, unique identifier, synonyms] |
| 2 | exp Breast Feeding/ |
| 3 | exp Lactation/ |
| 4 | breastfeeding.mp. [mp=title, book title, abstract, original title, name of substance word, subject heading word, floating sub-heading word, keyword heading word, organism supplementary concept word, protocol supplementary concept word, rare disease supplementary concept word, unique identifier, synonyms] |
| 5 | lactation.mp. [mp=title, book title, abstract, original title, name of substance word, subject heading word, floating sub-heading word, keyword heading word, organism supplementary concept word, protocol supplementary concept word, rare disease supplementary concept word, unique identifier, synonyms] |
| 6 | 2 or 4 |
| 7 | 3 or 5 |
| 8 | 6 or 7 |
| 9 | exp Blindness/ |
| 10 | blindness.mp. [mp=title, book title, abstract, original title, name of substance word, subject heading word, floating sub-heading word, keyword heading word, organism supplementary concept word, protocol supplementary concept word, rare disease supplementary concept word, unique identifier, synonyms] |
| 11 | 9 or 10 |
| 12 | Vision, Low/ |
| 13 | low vision.mp. [mp=title, book title, abstract, original title, name of substance word, subject heading word, floating sub-heading word, keyword heading word, organism supplementary concept word, protocol supplementary concept word, rare disease supplementary concept word, unique identifier, synonyms] |
| 14 | 12 or 13 |
| 15 | reduced vision.mp. [mp=title, book title, abstract, original title, name of substance word, subject heading word, floating sub-heading word, keyword heading word, organism supplementary concept word, protocol supplementary concept word, rare disease supplementary concept word, unique identifier, synonyms] |
| 16 | 14 or 15 |
| 17 | 11 or 16 |
| 18 | 8 and 17 |
| 19 | (breastfeeding adj5 (skill* or experience* or need* or requirement* or educat* or support* or knowledg*)).mp. [mp=title, book title, abstract, original title, name of substance word, subject heading word, floating sub-heading word, keyword heading word, organism supplementary concept word, protocol supplementary concept word, rare disease supplementary concept word, unique identifier, synonyms] |
| 20 | 17 and 19 |
| 21 | Mothers/ |
| 22 | 17 and 21 |
| 23 | ((mother* or woman or women or female parent or mum*) adj4 (blind* or visually impaired or low vision or amauroses or diminished vision)).mp. [mp=title, book title, abstract, original title, name of substance word, subject heading word, floating sub-heading word, keyword heading word, organism supplementary concept word, protocol supplementary concept word, rare disease supplementary concept word, unique identifier, synonyms] |
| 24 | 8 and 23 |
