# Supplementary material for: Breastfeeding Experiences of Mothers With Visual Impairment: A Scoping Review
Source: Matern Child Nutr. 2025 Jun 29;21(4):e70061. doi: 10.1111/mcn.70061 (PMC12454189; doi:10.1111/mcn.70061)
Supplement: Supplementary file 3 — Embase search strategy. [file MCN-21-e70061-s003.docx]

| **Embase** | |
| --- | --- |
| **#** | **Query** |
| 1 | exp breast feeding/ |
| 2 | breastfeeding.mp. [mp=title, abstract, heading word, drug trade name, original title, device manufacturer, drug manufacturer, device trade name, keyword heading word, floating subheading word, candidate term word] |
| 3 | exp breast feeding education/ |
| 4 | breastfeeding education.mp. [mp=title, abstract, heading word, drug trade name, original title, device manufacturer, drug manufacturer, device trade name, keyword heading word, floating subheading word, candidate term word] |
| 5 | 3 or 4 |
| 6 | exp blindness/ or exp cerebral blindness/ or exp leber congenital amaurosis/ or exp partial blindness/ or exp unilateral blindness/ |
| 7 | exp low vision/ |
| 8 | exp visual impairment/ |
| 9 | blindness.mp. [mp=title, abstract, heading word, drug trade name, original title, device manufacturer, drug manufacturer, device trade name, keyword heading word, floating subheading word, candidate term word] |
| 10 | low vision.mp. [mp=title, abstract, heading word, drug trade name, original title, device manufacturer, drug manufacturer, device trade name, keyword heading word, floating subheading word, candidate term word] |
| 11 | visual impairment.mp. [mp=title, abstract, heading word, drug trade name, original title, device manufacturer, drug manufacturer, device trade name, keyword heading word, floating subheading word, candidate term word] |
| 12 | 6 or 7 or 8 or 9 or 10 or 11 |
| 13 | 5 and 12 |
| 14 | 1 or 2 |
| 15 | 12 and 14 |
| 16 | exp mother/ |
| 17 | mother.mp. [mp=title, abstract, heading word, drug trade name, original title, device manufacturer, drug manufacturer, device trade name, keyword heading word, floating subheading word, candidate term word] |
| 18 | 16 or 17 |
| 19 | 15 and 18 |
